# Modeling effects of crop production, energy development and conservation-grassland loss on avian habitat

**DOI:** 10.1371/journal.pone.0198382

**Published:** 2019-01-09

**Authors:** Jill A. Shaffer, Cali L. Roth, David M. Mushet

**Affiliations:** 1 U.S. Geological Survey, Northern Prairie Wildlife Research Center, Jamestown, North Dakota, United States of America; 2 U. S. Geological Survey, Western Ecological Research Center, Dixon Field Station, Dixon, California, United States of America; Tanzania Fisheries Research Institute, UNITED REPUBLIC OF TANZANIA

## Abstract

Birds are essential components of most ecosystems and provide many services valued by society. However, many populations have undergone striking declines as their habitats have been lost or degraded by human activities. Terrestrial grasslands are vital habitat for birds in the North American Prairie Pothole Region (PPR), but grassland conversion and fragmentation from agriculture and energy-production activities have destroyed or degraded millions of hectares. Conservation grasslands can provide alternate habitat. In the United States, the Conservation Reserve Program (CRP) is the largest program maintaining conservation grasslands on agricultural lands, but conservation grasslands in the PPR have declined by over 1 million ha since the program’s zenith in 2007. We used an ecosystem-services model (InVEST) parameterized for the PPR to quantify grassland-bird habitat remaining in 2014 and to assess the degradation status of the remaining grassland-bird habitat as influenced by crop and energy (i.e., oil, natural gas, and wind) production. We compared our resultant habitat-quality ratings to grassland-bird abundance data from the North American Breeding Bird Survey to confirm that ratings were related to grassland-bird abundance. Of the grassland-bird habitat remaining in 2014, about 19% was degraded by crop production that occurred within 0.1 km of grassland habitats, whereas energy production degraded an additional 16%. We further quantified the changes in availability of grassland-bird habitat under various land-cover scenarios representing incremental losses (10%, 25%, 50%, 75%, and 100%) of CRP grasslands from 2014 levels. Our model identified 1 million ha (9%) of remaining grassland-bird habitat in the PPR that would be lost or degraded if all CRP conservation grasslands were returned to crop production. Grassland regions world-wide face similar challenges in maintaining avian habitat in the face of increasing commodity and energy production to sate the food and energy needs of a growing world population. Identifying ways to model the impacts of the tradeoff between food and energy production and wildlife production is an important step in creating solutions.

## Introduction

Birds perform a variety of supporting, provisioning, regulating, and cultural services as defined by the Millennium Ecosystem Assessment [1]. Thus, the preservation of avian biodiversity has numerous positive benefits to society. Birds are important culturally in arts and literature; recreationally to birdwatchers and hunters; and economically as pollinators, pest predators, seed dispersers, and nutrient cyclers [2]. However, for over two decades, ornithologists have been raising the alarm about the precipitous decline of grassland birds, driven primarily by the loss and degradation of habitat by anthropogenic means [3, 4]. Despite acknowledgment of the issue, grassland-bird habitat continues to be lost and degraded [5–7], and avian populations continue to decline [8].

The Prairie Pothole Region (PPR) of North America is home to 38 of the 41 species classified by Sauer et al. [8] as grassland birds. However, most of the grasslands that these species rely upon for habitat have been converted to alternate uses [5]. Two primary causes of contemporary habitat loss are crop production and energy development that result in grassland conversion and fragmentation [6, 9, 10]. Neither of these drivers, (i.e., crop production or energy development), are waning. Lark et al. [6] estimated that total net cropland area increased nationwide by 2.98 million acres from 2008 to 2012, with the greatest increases occurring in the PPR. The largest regional crude-oil-production growth through 2025 in the United States is expected to come from the Bakken formation in North Dakota, USA [11]. The International Energy Agency [12] forecasts that the largest growth in world power-generating capacity will be from renewable energies, with the United States the second-biggest market after China. Regionally, the states of North Dakota and South Dakota have abundant wind resources, routinely ranking in the top 20 wind-producing states [13, 14].

A primary cause of habitat degradation is the fragmentation of remaining expanses of grassland habitat. Habitat fragmentation refers to the reduction in area of some original habitat, a change in spatial configuration (that is, spatial arrangement), and an increasing distance between the patches of what remains, through the subdivision of continuous habitat into smaller pieces [15, 16]. Fragmentation, while increasing overall heterogeneity within a landscape, can decrease the heterogeneity of individual habitat types within blocks of remaining grasslands. With the loss of heterogeneity within grasslands comes an associated loss of biodiversity. Fragmentation also lowers habitat quality because of edge effects, such as lower avian reproductive success near the edge than the interior of remaining habitat [17]. The indirect effects on habitat quality can be much larger than the direct effects of habitat loss. For example, McDonald et al. [18] found that 5% of habitat impacts to grassland birds were due to the direct effects of land-clearing activities associated with natural gas and petroleum development, but 95% were the result of habitat fragmentation and species-avoidance behavior. For wind turbines, they found similar direct and indirect impacts, 3–5% direct and 95–97% indirect. Thus, any evaluation of grassland-bird habitats should include an assessment of the indirect effects on the quality of remaining habitats.

To offset the loss and degradation of native habitats, and the services they provide, both governmental and nongovernmental organizations have made significant monetary investments to restore and protect grassland habitats in the PPR. Given the prominence of agriculture throughout the PPR, the most wide-reaching conservation efforts have been associated with various programs of the U.S. Department of Agriculture (USDA). Within the USDA, the Conservation Reserve Program (CRP) has had the largest impact in terms of establishing perennial grasslands on areas previously used for crop production (S1 Table) [19]. These conservation grasslands provide numerous ecosystem services, including climate regulation, water purification, and erosion regulation [20]. Habitat created by the conservation grasslands is important in maintaining populations of wildlife, including grassland-bird species [21–24]. These conservation grasslands can also buffer other adjacent grasslands from the indirect effects of crop production and energy development activities. However, payments to agricultural producers participating in the CRP and other conservation programs have often failed to keep pace with rising values of agricultural commodities and land-rental rates [25]. The disparity of profits between participation in a conservation program versus production of agricultural commodities or the rental of land for crop production has resulted in a recent exodus of agricultural producers from conservation programs [6, 20, 26]. Since peak enrollment of 14.9 million ha in 2007, CRP grasslands have declined 25% nationally [20]. CRP grasslands in the four states comprising the PPR declined from more than 3.5 million ha in 2007 to just over 2.3 million ha in 2012, a 35% decline [27]. Additionally, new varieties of pesticide-tolerant and drought-resistant crops, as well as the rising popularity of corn (*Zea mays*) and soy (*Glycine max*) as biofuels, have resulted in the production of row crops in many areas previously dominated by small-grain production and conservation grasslands [27].

In addition to the current loss of conservation grasslands to crop production, increasing demand for domestic energy sources will likely have a negative impact on grassland quantity and quality. McDonald et al. [18] estimated that 20.6 million ha of new land will be required to meet U.S. energy demands by 2030, with temperate grasslands projected to be one of the most highly impacted terrestrial habitat types. The most intact grassland landscapes in the PPR are generally located on high-elevation geological features that are too rugged for mechanized agricultural equipment or too dry for row-crop agriculture, but even these grasslands are threatened due to their potential as sites for wind facilities or for oil and gas development [9, 10].

**Fig 1:**
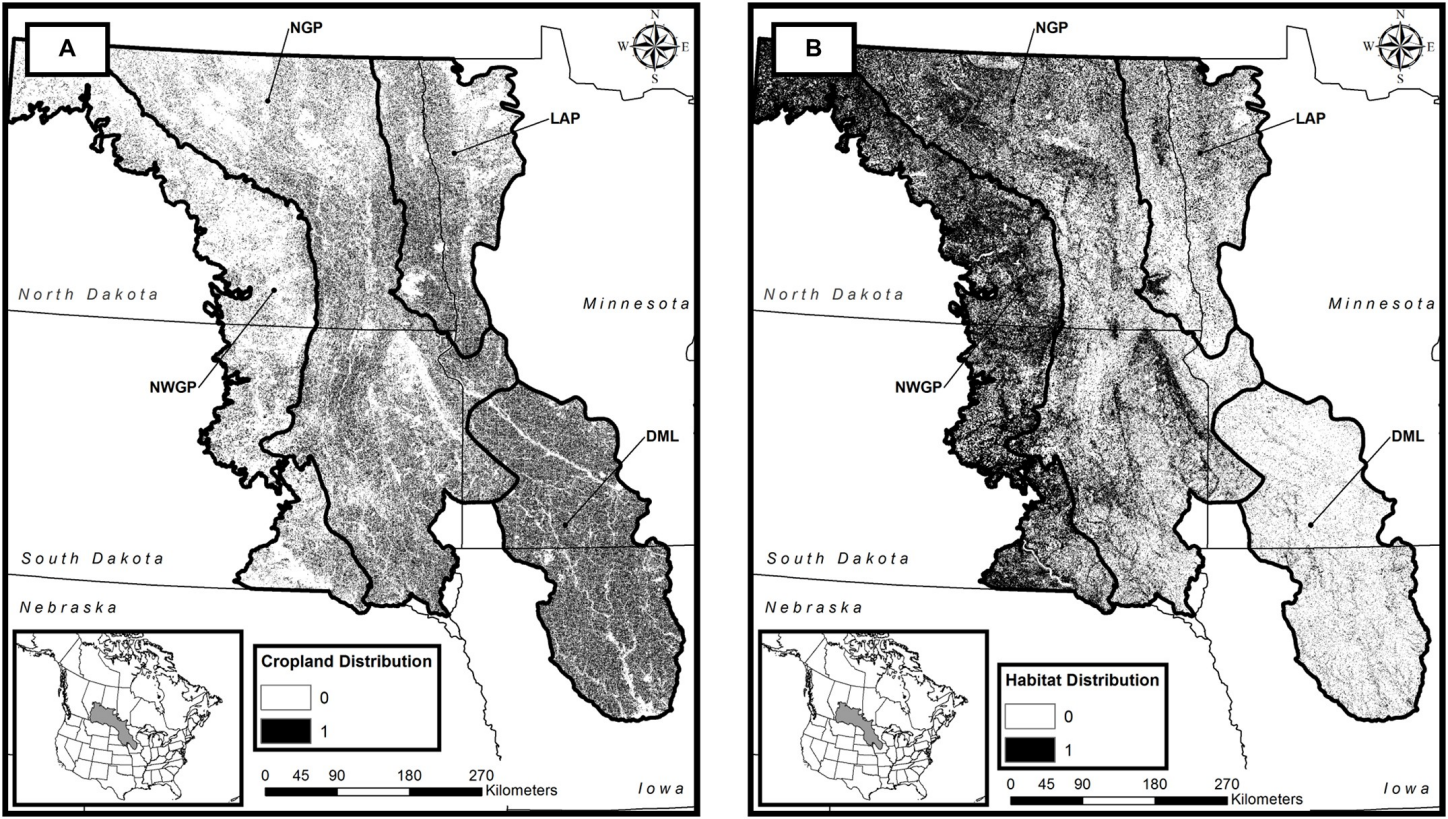
Distribution of cropland (Map A) and suitable grassland-bird habitat with an InVEST habitat-quality ranking ≥ 0.3 (indicated in black) (Map B) in the Prairie Pothole Region of the United States in 2014. Ecoregions are the Northern Glaciated Plains (NGP), Northwestern Glaciated Plains (NWGP), Lake Agassiz Plain (LAP), and Des Moines Lobe (DML) ecoregions [28].

To increase our understanding of how crop production and energy development has affected the integrity of avian habitat, we quantified suitable grassland-bird habitat across the three Level III ecoregions (Northern Glaciated Plains, Northwestern Glaciated Plains, and Lake Agassiz Plain) [28] and one level IV ecoregion (Des Moines Lobe) [28] that constitute the United States portion of the PPR (Fig 1). We did not attempt to quantify the impact of historic habitat losses in the PPR on grassland birds. Instead, we focused on the contemporary impacts that crop production and energy development activities have on remaining habitats and the role of conservation grasslands in mitigating these impacts. Our specific research objectives were to: 1) quantify the current (2014) grassland-bird habitat within the PPR using a modeling approach that incorporates indirect impacts to habitat integrity, 2) verify that the resultant habitat-quality rankings are related to grassland-bird abundance, 3) quantify the contribution of oil, natural gas, and wind development to the degradation of the remaining grassland habitat, and 4) quantify the habitat degradation that would occur if various percentages of CRP conservation grasslands in the PPR were returned to row-crop production. Recognizing that crop production and energy development will likely continue to cause loss and degradation of the remaining grassland-bird habitats, and that CRP grasslands continue to decline across the PPR, we provide a baseline scenario against which future habitat projections can be compared.

## Material and methods

### Study area

The PPR covers approximately 82 million ha of the United States and Canada (Fig 1). Glacial processes shaped the region and created a landscape consisting of millions of palustrine wetlands (often termed prairie potholes) interspersed within a grassland matrix [29, 30]. The PPR is recognized as one of the largest grassland/wetland complexes in the world [31]. It is a globally important ecosystem for a wide variety of flora and fauna including grassland and wetland plants [32], grassland birds [33], shorebirds [34], waterbirds [35], waterfowl [36], small mammals [37], amphibians [38], and aquatic and terrestrial invertebrates, including pollinators [30, 39, 40]. Despite the biological value of the PPR, grassland loss continues, and conservation efforts are not keeping pace [5, 6, 40, 41].

In addition to supporting grassland- and wetland-dependent biota, the combination of the region’s rich glacial soils and temperate climate has made it an ideal area for agricultural commodity production [42]. To facilitate crop production, approximately 95% of the native tallgrass prairie and 60% of native mixed-grass prairie have been converted to croplands since European settlement (Fig 1) [43]. In an effort to increase our understanding of how this land-cover change has affected the integrity of avian habitat, we quantified suitable grassland-bird habitat across the three Level III ecoregions (Northern Glaciated Plains, Northwestern Glaciated Plains, and Lake Agassiz Plain) [28] and one level IV ecoregion (Des Moines Lobe) [28] that constitute the United States portion of the PPR (Fig 1).

### Modeling approach

We used the Habitat Quality Module of the Integrated Valuation of Ecosystem Services and Tradeoffs (InVEST) modeling suite version 3.2.0 [44] to quantify grassland-bird habitat. InVEST is a suite of spatially based modeling tools that quantify services derived from ecosystems, including the maintenance of wildlife habitats [45]. Using InVEST, we modeled grassland-bird habitat for the year 2014. We chose 2014 because it is the most current year for which we could obtain both energy-development and CRP data layers. We created land-cover data layers by combining the 2014 National Agricultural Statistics Service (NASS) cropland data layer (raster, 30 m^2^) and a shape file obtained from USDA Farm Service Agency’s Economics and Policy Analysis Staff that identified areas enrolled in the CRP in 2014. A complete description of our development of the land-cover layers used in InVEST runs is provided online in S2 Table.

To develop a baseline habitat layer, we assigned habitat suitability weights from 0–1 to each land-cover pixel in the cropland data layer (overall accuracy > 83%). Weights were assigned relative to one another, with higher weights representing the most suitable habitat. Suitable grassland-bird habitat was defined as any land-cover category of grassland (i.e., herbaceous grassland [e.g., native prairie], CRP grassland, hayland) and specific categories of small-grain cropland (S3 Table). For example, native prairie and CRP grassland were equally highly weighted (i.e., 1.0), small-grain cropland received a weight half that of grasslands (i.e., 0.5), fallow land received the lowest weight for habitats (i.e., 0.3), and non-habitat land-cover classes received a weight of 0. For our analysis, suitable grassland-bird habitat was defined as any pixel with a habitat rating ≥ 0.3, (i.e., the lowest weight assigned to a land-cover class identified as habitat). InVEST takes habitat models one step beyond relative habitat-suitability rankings by incorporating threats to habitat integrity, weighting those threats relative to one another, incorporating the linear distance that those threats influence adjacent habitats, and ranking the sensitivity of habitats to each threat. We identified threats to grassland-bird habitat as the primary causes of fragmentation and degradation of large tracts of grasslands. The primary habitat threats identified were: 1) woodland, 2) urbanization, 3) cropland, 4) roads, and 5) energy development [5, 46–54]. We weighted each threat from 0–1 by expected impact to grassland-bird habitat, with higher weights representing greater habitat degradation (S4 Table). We determined the distance that threats acted upon nearby habitats based on published literature [9, 10, 47, 48, 50, 51, 55, 56]. We used InVEST to apply these threats to our baseline habitat raster to account for their degradation of nearby habitat.

We assigned the greatest threat value to woodland and urbanized areas because grassland birds find these land-cover types virtually unsuitable for all aspects of their life cycle and they harbor predators and nest parasites that affect quality of nearby habitats [17]. Cropland can serve as habitat, (e.g., grains and berries serve as food sources and vegetation serves as escape and shade cover), but disturbance associated with weed control, tillage and harvest usually precludes successful nesting, if nesting is even attempted [57]. Roads, well pads and turbine pads accompanying energy development generally have a small relative footprint on a landscape level, and species show varying degrees of tolerance to these types of disturbances [9,10].

At a pixel level in the InVEST model, a pixel’s original habitat-ranking value can decrease because of its proximity to a threat, causing one of two outcomes: a decrease in value such that the pixel no longer maintains a value ≥ 0.3, (i.e., is lost as suitable habitat), or a decrease in value but not below 0.3, (i.e., a degradation in quality but still available as suitable habitat). Thus, loss of habitat can occur under two situations: 1) when a pixel becomes converted from a habitat land-use category to a non-habitat category, as in the situation whereby native prairie gets converted to corn, or 2) when a pixel itself does not change land-use category, but a change in a nearby pixel triggers the threat distance to decrease the focal pixel’s value below 0.3. Subsequently, we chose to isolate and examine the impact of two of our five threats, cropland and energy development, because cropland has the greatest footprint in the PPR (Fig 1A) and is the traditional and ongoing major cause of habitat loss for grassland birds, whereas energy development is a more recent, but still developing, threat, and its impact is more localized.

We created binary rasters of each threat’s location across the PPR. We developed cropland and woodland threat layers through a reclassification process of land-cover layers using R (version 3.2.0, packages rgdal, raster, sp, and rgeos) [58]. We developed urban and road threat layers using a combination of 2015 Tiger/Line city census data and NASS and developed the energy threat layer by downloading 2014 locations publicly available through the U.S. Geological Survey (S2 Table). We buffered the turbine locations by 30 m [59] and the gas and oil well locations by 100 m [9] to represent surface impact. When threat locations were applied to the landscape in the model, every threat’s weight decayed linearly over the maximum distance of its impact, representing greater impact at closer proximity to the threat.

To verify that habitat-quality scores are positively associated to grassland-bird abundance, we related the habitat-quality scores output by the model to breeding-bird abundance data using negative binomial regression due to the over-dispersed nature of the count data [60]. We validated the use of InVEST to calculate our habitat covariate by comparing the performance of the InVEST habitat model (*InVEST*) to an intercept-only model (*Null*) and a baseline habitat rank model (*Baseline*). We based our bird-abundance estimates on ten avian species that represent mixed-grass prairie endemics and that are considered grassland birds as categorized by Sauer et al. [8]: upland sandpiper (*Bartramia longicauda*), Sprague’s pipit (*Anthus spragueii*), chestnut-collared longspur (*Calcarius ornatus*), clay-colored sparrow (*Spizella pallida*), savannah sparrow (*Passerculus sandwichensis*), vesper sparrow (*Pooecetes gramineus*), grasshopper sparrow (*Ammodramus savannarum*), Baird’s sparrow (*Ammodramus bairdii*), bobolink (*Dolichonyx oryzivorus*), and western meadowlark (*Sturnella neglecta*). We acquired count data for these species from the North American Breeding Bird Survey (BBS), a continental, road-side survey conducted annually since 1966 [8, 61]. We pooled the sum of the counts of all ten species from 2013–2015 (N = 2100) by BBS stop for North Dakota, the state for which spatial coordinates by stop were available [62]. We included the years on either side of 2014 to capture the full temporal shift in bird response to disturbance caused by initial development of threats as well as potential temporal lags in grassland-bird responses to threat establishment, respectively. We buffered each survey stop by 400 m, the distance at which birds are assumed to be detected in the surveys and calculated the mean habitat quality within this buffer from our InVEST output and compared these values to the grassland-bird abundance estimate for that point.

We next used InVEST to quantify current (2014) grassland-bird habitat quality and quantity, and grassland-bird habitat quality and quantity among our various scenarios of CRP loss for the PPR within the United States. For our CRP grassland loss scenarios, we created polygon sets containing 100%, 75%, 50%, 25%, 10% and 0% of the CRP fields in our 2014 baseline land-cover layer using a random, successive subsetting method so that CRP fields included in lower percentage sets were also included in the higher percentage sets. Using each set of polygons as a mask, these fields were converted to row crops in our baseline land-use layer to simulate the conversion of CRP grassland habitat to agriculture. By removing percentages of fields rather than total area in our baseline data layer, we followed the assumption that if an agricultural producer decided to remove land from a conservation program, this decision would be made on a field-by-field basis rather than on an unrealistic pixel-by-pixel basis. We compared land-cover layers for each percentage-loss scenario to total CRP grassland area in the 0% loss layer to verify that the correct percentage of CRP grassland was converted to cropland. We used an output cell size of 30 m. A half-saturation constant of 0.20 was selected by comparing multiple runs of the InVEST model and was used to optimize visual display of the resulting layer [63]. In each run (i.e., scenario), the model worked to erode the quality value of identified grassland-bird habitats (initial value ≥0.3) based on spatial proximity to a threat, susceptibility to that threat, and the threat’s strength (i.e., threat weight). Output data layers from the model were used to create maps depicting changes in grassland-bird habitat quality among scenarios of CRP loss. From our habitat quality maps, we produced summary tables quantifying changes in suitable-habitat quantity (ha) by ecoregions.

## Results

**Fig 2:**
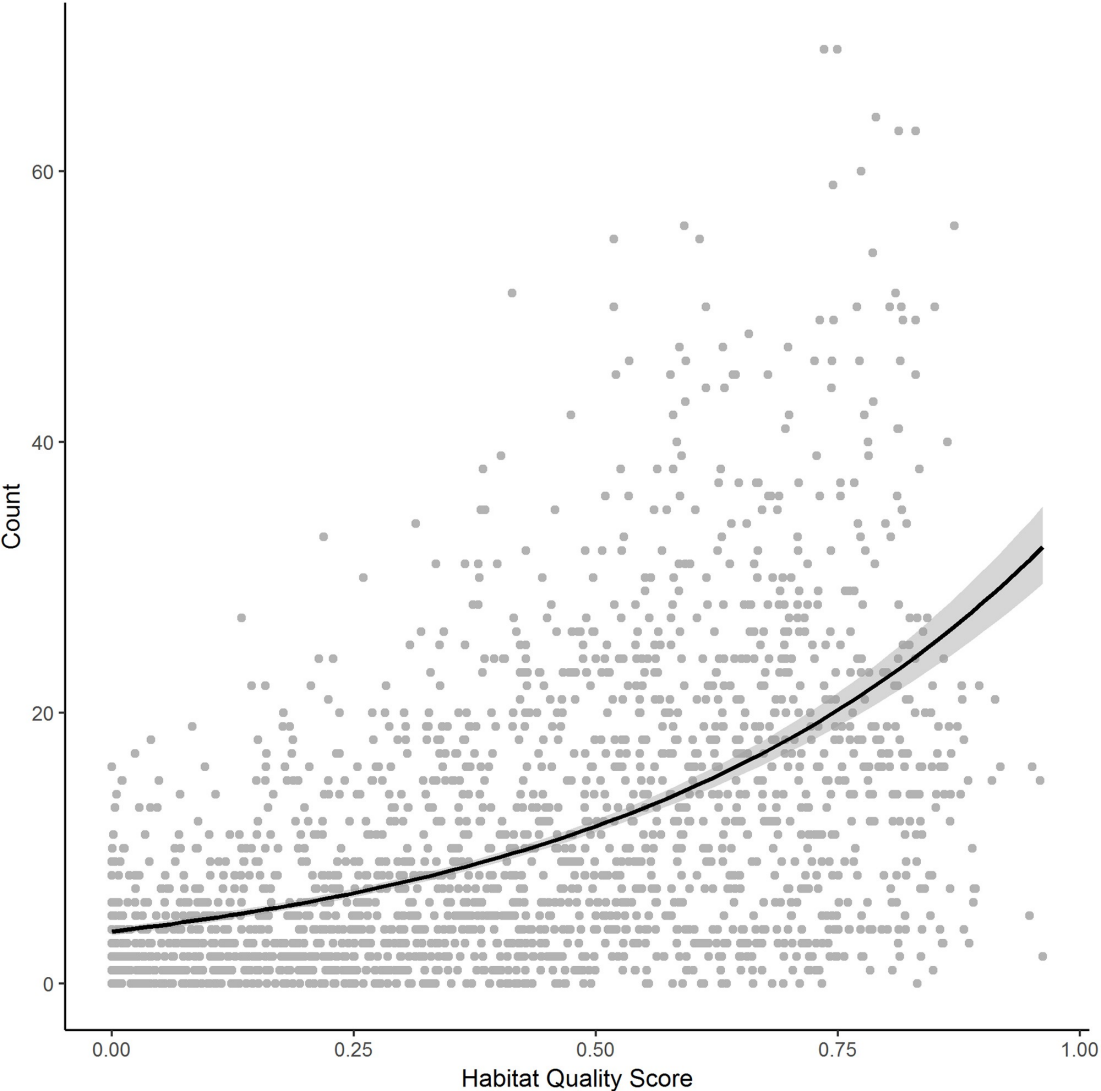
Scatter plot of habitat-quality rating versus bird abundance (with 95% confidence interval band) for 2100 points surveyed during the 2013–2015 North American Breeding Bird Survey.

Compared to the intercept-only model and the baseline model, the InVEST habitat model better accounted for increases in breeding-bird abundance (ΔAIC > 2; Table 1). We verified that resultant InVEST habitat-quality ratings were positively related to abundance of grassland birds in North Dakota (coefficient = 1.76, ±97.5% C.I. = 0.15, Fig 2). The relationship between abundance estimates from BBS surveys and our modeled bird abundance was significantly different from zero (C.I. range: 1.61–1.92). We calculated a pseudo R-squared of 0.29 (±97.5% C.I. = 0.03), indicating fair model fit but suggesting that unmeasured covariates in addition to habitat quality influenced actual bird occurrence. Also, of note, BBS stops with a habitat score < 0.3 had an average abundance of 5, and those with scores ≥ 0.30 had an average abundance of 15. Thus, while points with high habitat-quality ratings were associated with both low and high bird abundance, points with low quality ratings were almost always associated with low bird abundance (Fig 2).

**Table 1:** Results of model selection among intercept-only, baseline-habitat score, and InVEST habitat-score models.

Response	Model	K	AICc	Weight	ΔAICc	LogLikelihood
BBS Counts	InVEST*	4	12368.18	1	0	-6180.08
	Baseline	4	12375.95	0.02	7.77	-6183.96
	Null	3	12852.43	0	484.25	-6423.21

*Selected model

From our baseline (2014) model and our definition of suitable habitat as any land-cover type with a habitat-quality ranking higher than 0.3, we estimated that around 12 million ha of suitable grassland-bird habitat (i.e., habitat quality score ≥0.3) remained within the four PPR ecoregions in 2014 (Table 2; Fig 1B). The Northern Glaciated Plains and Northwestern Glaciated Plains ecoregions accounted for over 80% of the suitable grassland-bird habitat. Availability of suitable grassland-bird habitat was lowest in the Des Moines Lobe ecoregion. The area of cropland (8.9 million ha) greatly exceeded the area devoted to energy development (44.5 thousand ha, Table 2).

**Table 2:** Area (ha) of suitable (i.e., a relative habitat-quality ranking ≥ 0.3 out of a maximum value of 1.0) grassland-bird habitat and of non-suitable habitat that was devoted to cropland and energy development in 2014 within the Northern Glaciated Plains (NGP), Northwestern Glaciated Plains (NWGP), Lake Agassiz Plain (LAP), and Des Moines Lobe (DML) ecoregions of the United States. Areas were quantified using the National Agricultural Statistics Service Cropland Data Layer.

Ecoregion	Grassland-bird Habitat	Non-habitat Cropland	Energy Development Land
NGP	5,256,338	3,571,532	22,502
NWGP	4,751,716	980,650	21,290
LAP	1,070,396	1,350,374	3
DML	457,953	3,015,641	799
**Total**	**11,536,402**	**8,918,197**	**44,595**

Our application of the InVEST model to quantify effects of cropland and energy development demonstrated low impact (65,800 ha) in causing original habitat-quality rankings to become unsuitable, (i.e., falling below 0.3) due to the influence of nearby cropland or energy development threats (Table 3). However, cropland and energy development had a much greater impact in terms of degrading the quality of habitat when habitats that did not drop below a score of 0.3 are included. In this case, cropland degraded 19% (2.1 million ha) of the available grass-land bird habitat, whereas energy development degraded 16% (1.9 million ha, Table 3). Among ecoregions, remaining grassland-bird habitats in the Northern Glaciated Plains and the Des Moines Lobe were degraded the most by cropland and the Northwestern Glaciated Plains the least, whereas the Northern Glaciated Plains and the Northwestern Glaciated Plains were degraded the most and the Des Moines Lobe the least by energy development. Although not nearly as ubiquitous in distribution as cropland, where energy development occurs, its localized impact can be significant (S1 Fig). Land within the PPR is surveyed according to the Public Land Survey System of dividing land into parcels, one division of which is a township comprised of thirty-six 1-mi^2^ (259 ha) sections [64]. We found entire townships were rendered unsuitable habitat by the clustering of oil wells in close proximity (S1 Fig). Our scenario quantifying the impact of cropland on the suitability of current (2014) CRP conservation grassland as grassland-bird habitat showed suitable habitat loss of less than 1%, although it caused degradation of 13% of the grassland-bird habitat (Table 3). The largest decline in habitat quality occurred in the Northern Glaciated Plains and the least in the Des Moines Lobe.

**Table 3:** Model results of the area (ha) of suitable grassland-bird habitat lost and degraded in four ecoregions of the United States under three threat scenarios: 1) influence of cropland, 2) influence of energy development, and 3) impact on Conservation Reserve Program (CRP) habitat value based on cropland threat. Baseline suitable habitat was quantified using the National Agricultural Statistics Service (NASS) Cropland Data Layer for 2014. Lost habitat indicates suitable habitat that fell below the relative habitat-quality rating of 0.3 on a maximum-scale value of 1.0. Degraded habitat indicates suitable habitat that dropped in habitat-quality ranking but stayed above 0.3 (i.e., was not lost). Values in parentheses represent the percentage of current (2014) suitable habitat degraded under the different scenarios. The ecoregions are the Northern Glaciated Plains (NGP), Northwestern Glaciated Plains (NWGP), Lake Agassiz Plain (LAP), and Des Moines Lobe (DML).

	NASS 2014	Application of the Habitat Quality Module of InVEST								
		Scenario 1: Cropland Threat			Scenario 2: Energy Threat			Scenario 3: Threat to CRP value by Cropland		
	Suitable Grassland Bird Habitat	Habitat that became unsuitable (lost) due to cropland threat	Suitable habitat degraded by cropland threat	Grassland bird habitat remaining	Habitat that became unsuitable (lost) due to energy threat	Suitable habitat degraded by energy	Grassland bird habitat remaining	Habitat that became unsuitable (lost) due to loss in CRP value	Suitable habitat degraded by impact of cropland on CRP value	Grassland Bird Habitat Remaining
NGP	5,256,338	1,784	1,131,551 (-21%)	**5,304,588**	29,188	1,011,304 (-19%)	**5,277,184**	265	835,229 (-16%)	**5,306,107**
NWGP	4,751,716	617	605,376 (-13%)	**4,783,109**	29,883	732,798 (-15%)	**4,753,843**	84	505,944 (-11%)	**4,783,642**
LAP	1,070,396	936	228,064 (-18%)	**1,244,091**	6	125,821 (-10%)	**1,245,021**	76	137,199 (-11%)	**1,244,951**
DML	457,953	2,644	183,393 (-31%)	**587,968**	0.8	20,800 (-4%)	**589,812**	526	24,994 (-4%)	**590,086**
**Total**	**11,536,402**	**5,981**	**2,148,384 (-19%)**	**11,919,756**	**59,877**	**1,890,723 (-16%)**	**11,865,860**	**951**	**1,503,366 (-13%)**	**11,904,786**

**Fig 3:**
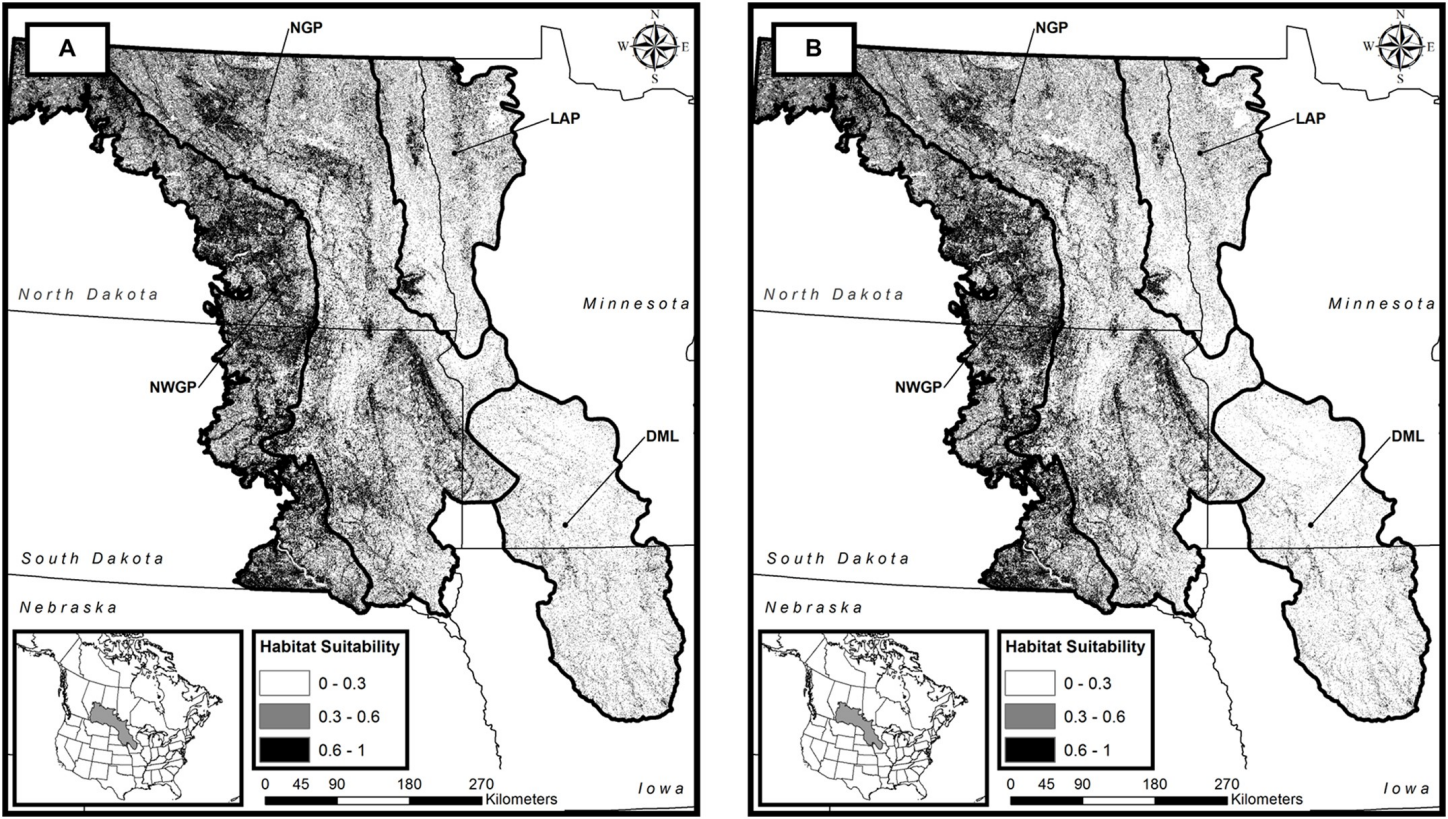
Distribution of suitable habitat with an InVEST habitat-quality ranking ≥ 0.3 under a scenario in which all Conservation Reserve Program (CRP) grasslands present in the Prairie Pothole Region of the United States in 2014 are intact (Map A) and a scenario in which all CRP grasslands are converted to row-crop production (Map B).

Our scenario-based CRP modeling revealed a loss in suitable grassland-bird habitat (-2% across the PPR) if 25% of CRP grasslands present in 2014 are returned to agricultural production. This loss of suitable habitat increases to 9% (a loss of approximately 1 million ha) if all CRP grasslands within the PPR are returned to agricultural production (Table 4; Fig 3A and 3B). Our modeling also reveals that the Des Moines Lobe would have the greatest relative loss of suitable grassland-bird habitat (-36% in our scenario in which all CRP grasslands are converted to cropland) and the Northwest Glaciated Plain the least at 3% (Table 4; Fig 3A and 3B).

**Table 4:** Area (ha) of suitable grassland-bird habitat with a relative habitat-quality ranking ≥ 0.3 on a maximum-scale value of 1.0 in the Northern Glaciated Plains (NGP), Northwestern Glaciated Plains (NWGP), Lake Agassiz Plain (LAP), and Des Moines Lobe (DML) ecoregions of the United States in the baseline year of 2014 and under five scenarios reflecting the conversion of 10%, 25%, 50%, 75%, and 100% of Conservation Reserve Program (CRP) grasslands to row crops. Values in parentheses represent the percentage of current (2014) suitable habitat lost under the different scenarios of CRP conversion.

		Scenarios				
	Current (2014)	-10% CRP	-25% CRP	-50% CRP	-75% CRP	-100% CRP
NGP	5,256,073	5,201,350 (-1%)	5,117.941 (-2.6%)	4,982,635 (-5.2%)	3,849,494 (-7.7%)	4,713,048 (-10.3%)
NWGP	4,751,631	4,736,025 (-0.3%)	4,713,506 (-0.8%)	4,675,765 (-1.6%)	4,635,920 (-2.4%)	4,597,735 (-3.2%)
LAP	1,070,319	1,049,800 (-1.9%)	1,019,354 (-4.8%)	968,130 (-9.6%)	915,492 (-14.5%)	865,272 (-19.2%)
DML	457,427	440,827 (-3.6%)	415,275 (-9.2%)	373,533 (-18.3%)	332,627 (-27.3%)	291,988 (-36.2%)
**Total**	**11,535,451**	**11,428,001 (-0.9%)**	**11,266,075 (-2.3%)**	**11,000062 (-4.6%)**	**10,733,532 (-7.0%)**	**10,468,042 (-9%)**

## Discussion

We demonstrated both the utility of applying the InVEST-modeling approach to quantifying habitat suitability for grassland birds and for estimating the effects of land-cover conversion scenarios on these habitats. An important distinction between InVEST and other approaches is that InVEST allows for not only the modeling of land-cover conversion scenarios, but also the quantification of how habitat “threats” impact landscape-level habitat availability to an organism. This allows for more robust quantifications of how matrices of land cover, some of which are suitable habitat for birds and some of which are habitat threats, interact to affect overall landscape integrity, in our case for grassland birds.

We did not attempt to forecast grassland-bird population sizes, but rather quantified habitat quality as influenced by threats and susceptibility to those threats. Multiple factors in addition to summertime nesting habitat affect grassland-bird populations; some (e.g., condition of wintering habitat) are far removed from our study region. Thus, prediction of population sizes was beyond the scope of our work. However, habitat-quality information derived from the methodology described here could play an important role in the development and improvement of grassland-bird population models. We also did not attempt to quantify a monetary aspect to the losses in grassland-bird habitat. While the estimation of monetary gains or losses associated with loss or degradation of habitats and effects on ecosystem services is useful in guiding decisions, such quantifications were well beyond the scope of our research effort. Rather, we focused on the preliminary step necessary to calculate the monetary effects of losses and degradation, that being the quantification of habitat losses and degradation itself. We provide methodology to obtain such quantifications. Additionally, in most cases, monetary gains or losses resulting from losses and degradation of habitat need to be calculated on a case-by-case basis as each “project” has its own, often very different, inputs in terms of costs (e.g., converting land, installing wind turbines, constructing roads, loss of birding opportunities, degradation of other ecosystem services) and benefits (e.g., food production, energy production, restoration of bird populations).

To illustrate our method, we chose to quantify the degree to which one traditional and widespread threat, cropland, and one nascent but more localized threat, energy development, influenced the availability of suitable grassland-bird habitat in the current (2014) matrix of land cover in the PPR. We further illustrate the differences that these two threats have to a specific area. It is key to note that, with the exception of our CRP-conversion scenarios, we did not quantify the direct loss of habitat resulting from conversion of grasslands to cropland or due to energy development. Rather, we quantified the effects of habitat threats within the current (2014) landscape configuration on the remaining area of suitable grassland-bird habitat within that landscape. Because of cropland’s pervasiveness throughout the PPR, its cumulative impact as a threat to remaining grassland-bird habitat is great, degrading remaining grassland-bird habitat at rates varying from 13–31% across the region (Table 3). Energy development, as a much more localized threat, had a smaller impact at 4–19% degradation rates across the region. However, in places where energy development has occurred, the localized impact has affected entire blocks of 36 mi^2^ (93.2 km^2^) townships (S1 Fig). By examining these threats at the ecoregion level, we were able to determine those ecoregions in which grassland-bird habitats have been the most impacted by either cropland or energy development.

Cropland and energy development threats caused <1% of remaining grassland-bird habitat to fall from “suitable” to “unsuitable” as habitat. This may be explained in terms of where cropland and energy development occur, which is in rural areas where, when a land-cover change occurs (i.e., a crop/non-crop interface), that other edge is most likely to be grassland, which will have a fairly high relative suitability ranking. The impact to watch, therefore, is the degree to which remaining suitable habitat is degraded due to its proximity to cropland and energy development. It is in this category that we see the influence of cropland and energy take a marked toll on the integrity of grassland-bird habitat. It is also important to note that not all cropland areas are unsuitable as grassland-bird habitat. Grassland-like crops and small-grains, such as alfalfa and wheat, have some value as avian habitat, whereas row crops such as corn and soybeans do not (S3 Table). Therefore, we would expect highest degradation in highly fragmented areas, (e.g., where grassland and cropland edges regularly abut, and where those cropland edges are row crops). The highest degradation, 31%, occurred in the Des Moines Lobe, which includes the extensive corn and soy fields of Iowa. A final point is that the low amount of habitat that fell below 0.3 indicates that the greatest threat to grassland integrity is not degradation, but the more direct effects of conversion to row crops, in which pixels that rank as high as 1 immediately fall below 0.3 upon conversion.

As to energy development, the largest congregation of oil and gas wells in the PPR is in the Bakken Region of northwestern North Dakota, and it is in the Northern Glaciated Plains that energy development has caused the greatest degradation in remaining grassland-bird-habitat quality. The threat of cropland to CRP habitat quality is fairly uniform across all ecoregions except the Des Moines Lobe, which has minimal degradation, which would occur if very little CRP occurred in that ecoregion. In ecoregions in which CRP is a large component of the grassland landscape, its adjacency to cropland threatens its integrity. In these areas, maintaining primarily grassland landscapes, either of CRP or native prairie, will be important for the maintenance of grassland-bird-habitat quality.

Our application of InVEST’s Habitat Quality Module to the CRP-conversion scenario revealed that if all-remaining CRP lands are returned to crop production, losses of suitable grassland-bird habitat would equal approximately 9% of the total suitable habitat available across the PPR in 2014. The CRP is a long-acknowledged driver in the maintenance and stabilization of grassland-bird populations [65–67]. The effects on grassland birds of losing close to one-tenth of their remaining suitable habitat in the PPR would undoubtedly be significant, and each ecoregion would face unique circumstances. The Des Moines Lobe and Lake Agassiz Plain ecoregions have already lost most of their natural grassland habitat due to intensive agricultural development. The Des Moines Lobe, which would lose 36% of its remaining suitable grassland-bird habitat, and the Lake Agassiz Plain, which would lose 19%, can each barely afford to lose additional habitat. Even with CRP intact, several grassland-bird species in these regions are in decline and species of federal conservation concern [68]. The loss of CRP could plausibly facilitate the extirpation of several grassland-bird species and render those regions species depauperate.

The Northern and Northwestern Glaciated Plains each have significantly more remaining grassland-bird habitat than the other two ecoregions. However, our model results demonstrate that loss of CRP would affect them at different levels; amount of suitable habitat in the Northern Glaciated Plains (10% loss of grassland-bird habitat under 100% CRP loss scenario) was more dependent on CRP lands than in the Northwestern Glaciated Plains (3% loss under the same CRP loss scenario). Most of the Northwestern Glaciated Plains is made up of an area known as the Missouri Coteau. The topography of the Missouri Coteau is varied, with greater local relief and rockier, less fertile, soils than in the Northern Glaciated Plains to the east. As a result, croplands, while still the major land cover-type, are less abundant, and native grassland pastures form a larger component of the Northwestern Glaciated Plains landscape than do conservation grasslands. CRP grasslands still provide significant habitat in this ecoregion, but native pastures also contribute to the maintenance of the ecoregion’s avian biodiversity. Even so, loss of CRP grasslands in the Northwestern Glaciated Plains are compounded by the impact of oil and gas development prevalent in this region and likely have a negative impact on species of conservation concern, such as the Sprague’s Pipit, Baird’s Sparrow, and McCown’s Longspur (*Rhyncophanes mccownii*) [67].

The results of our modeling efforts identify recent past and potential future bird habitat losses in the PPR of the United States. However, they also identify opportunities for the improvement of habitats if current trends can be reversed, either through gains in CRP or through other conservation programs that lead to increases in grassland habitats on the PPR landscape (e.g., USDA Natural Resources Conservation Service’s Agricultural Conservation Easement Program). The potential of conservation grasslands to mitigate grassland-bird habitat loss in the PPR has been demonstrated by the amount of suitable habitat that has been created on the landscape through a single conservation program, the CRP. If the CRP was not as successful as it has been in providing avian habitat on the PPR landscape, we would not see losses of these lands from the landscape resulting in such significant declines in suitable grassland-bird habitat in our modeled scenarios, and our validation work demonstrated that declines in habitat-quality ratings are related to declines in overall grassland-bird abundances. Thus, the CRP and other conservation programs can play a significant role in restoring grassland-bird populations in the PPR. However, care must be taken to recognize the transitory nature of conservation lands that are not protected through fee-title ownership or through long-term easements. As seen through recent losses of CRP conservation grasslands across the PPR landscape, lands protected through short-term contracts will likely revert to other uses during periods when conservation payments lag behind profits that can be realized through conversion back to crop production. The CRP is subject to shifting political priorities. In recent years, the CRP enrollment cap as enacted by the U.S. Congress has fallen from the peak of 14.9 million hectares in 2007 to 9.7 million hectares in 2018 and may slightly rise to 11 million hectares with the passage of the 2018 Farm Bill. Although the value of the CRP to grassland birds is irrefutable, developing other programs to sustain grassland-bird populations could provide a buffer to the transitory nature of CRP grasslands. Populations of grassland birds would be expected to rise with increasing CRP conservation grasslands but maintaining that population growth when CRP conservation grasslands are converted back to cropland if Farm Bill caps again drop will be a challenge without the existence of grasslands created in other programs.

An economic climate driven by demands for commodities has resulted in marked losses of grassland-bird habitat not just in the PPR, but worldwide. The resulting impact on species dependent upon habitat provided by natural and conservation lands could be substantial as these lands are converted to commodity production. Conversely, providing perennial grassland cover on agricultural lands through conservation programs has great potential to reverse these trends. Our results are applicable beyond the PPR in areas where grassland-bird habitats consist of grasslands embedded in a cropland matrix and economic pressures favor the conversion of natural and conservation grasslands to crop production and energy development. The application of scenario-based models such as InVEST to quantify grassland-bird habitats can identify potential regional effects of land-cover change. This knowledge will be needed to facilitate the improvement and ultimate success of grassland-bird conservation efforts.

## Supporting information

S1 Table Area (ha) of land within Minnesota (MN), North Dakota (ND), South Dakota (SD), and Iowa (IA) enrolled in the U.S. Department of Agriculture’s Conservation Reserve Program, 2007 to 2014 (USDA 2016). (DOCX)

S2 Table Sources of information for all spatial layers used to model grassland-bird habitat in the Prairie Pothole Region of the United States. (DOCX)

S3 Table InVEST habitat and sensitivity table. Rankings of National Agricultural Statistics Service land-cover habitat categories by suitability as breeding habitat for grassland-bird species (Habitat column) and ranking of sensitivity of those habitat categories to each of five threats to grassland-bird species in the Prairie Pothole Region of the United States.
(DOCX)

S4 Table InVEST threat table. Land-use categories treated as threats to the integrity of grassland-bird habitat in the Prairie Pothole Region of the United States are organized by their relative threat value, or weight. Distance reflects how far an influence a pixel of a threat exerts on surrounding pixels.
(DOCX)

S1 Fig Distribution of unsuitable habitat due to the impact of oil development in the Bakken Region of northwestern North Dakota, United States of America, showing the negative impact on habitat suitability of oil wells, the black squares. (DOCX)
