# Supplementary material for: Modeling effects of crop production, energy development and conservation-grassland loss on avian habitat
Source: PLoS One. 2019 Jan 9;14(1):e0198382. doi: 10.1371/journal.pone.0198382 (PMC6326430; doi:10.1371/journal.pone.0198382)
Supplement: S1 Table — (DOCX) [file pone.0198382.s001.docx]

**Supporting information**

**S1 Table**

Area (ha) of land within Minnesota (MN), North Dakota (ND), South Dakota (SD), and Iowa (IA) enrolled in the U.S. Department of Agriculture’s Conservation Reserve Program, 2007 to 2014 (USDA 2016).

| State | 2007 | 2008 | 2009 | 2010 | 2011 | 2012 | 2013 | 2014 | Change  2007 to 2014 | % Change  2007 to 2014 |
| --- | --- | --- | --- | --- | --- | --- | --- | --- | --- | --- |
| MN | 740,918 | 718,466 | 686,395 | 666,479 | 634,496 | 568,693 | 558,565 | 525,903 | -215,015 | -29% |
| ND | 1,372,332 | 1,205,433 | 1,155,257 | 1,076,375 | 969,053 | 730,595 | 720,741 | 655,315 | -717,017 | -52% |
| SD | 631,534 | 527,196 | 505,804 | 467,274 | 446,761 | 396,895 | 393,476 | 377,397 | 254,137 | -40% |
| IA | 798,047 | 732,827 | 690,092 | 680,700 | 671,500 | 620,078 | 617,151 | 589,837 | 208,210 | -26% |
| Total | 3,542,831 | 3,183,992 | 3,037,548 | 2,890,827 | 2,721,809 | 2,316,191 | 2,289,933 | 2,148,452 | 1,394,379 | -39% |
