# Supplementary material for: Modeling effects of crop production, energy development and conservation-grassland loss on avian habitat
Source: PLoS One. 2019 Jan 9;14(1):e0198382. doi: 10.1371/journal.pone.0198382 (PMC6326430; doi:10.1371/journal.pone.0198382)
Supplement: S2 Table — (DOCX) [file pone.0198382.s002.docx]

**Supporting Information**

**S2 Table**

Sources of information for all spatial layers used to model grassland-bird habitat in the Prairie Pothole Region of the United States.

| **Layer** | **Source Agency** | **Access Address** |
| --- | --- | --- |
| Cropland Data Layer (CDL) | National Agricultural Statistics Survey; US Department of Agriculture | https://nassgeodata.gmu.edu/CropScape/ |
| Conservation Reserve Program | Natural Resource Conservation Service; US Department of Agriculture | Not available for public download |
| Cropland Threat (CDL) | National Agricultural Statistics Survey; US Department of Agriculture | <https://nassgeodata.gmu.edu/CropScape/> |
| Woodland Threat (CDL) | National Agricultural Statistics Survey; US Department of Agriculture | <https://nassgeodata.gmu.edu/CropScape/> |
| Urbanization Threat (CDL) | Tiger/Line Urban Centers US Census Bureau  National Agricultural Statistics Survey; US Department of Agriculture | <https://www.census.gov/geo/maps-data/data/tiger-line.html>, <https://nassgeodata.gmu.edu/CropScape/> |
| Energy Development Threat | US Geological Survey | Wind Turbines: http://pubs.usgs.gov/ds/817/  Gas/Oil Wells: <https://www.sciencebase.gov/catalog/item/545d0446e4b0ba8303f71a55> |
| Roads Threat | Tiger/Line All Roads (available by county) US Census Bureau | https://www.census.gov/geo/maps-data/data/tiger-line.html |
| BBS data | US Geological Survey | https://www.pwrc.usgs.gov/bbs/results/ |
