# Supplementary material for: Modeling effects of crop production, energy development and conservation-grassland loss on avian habitat
Source: PLoS One. 2019 Jan 9;14(1):e0198382. doi: 10.1371/journal.pone.0198382 (PMC6326430; doi:10.1371/journal.pone.0198382)
Supplement: S3 Table — Rankings of National Agricultural Statistics Service land-cover habitat categories by suitability as breeding habitat for grassland-bird species (Habitat column) and ranking of sensitivity of those habitat categories to each of five threats to grassland-bird species in the Prairie Pothole Region of the United States. (DOCX) [file pone.0198382.s003.docx]

**Supporting Information**

**S3 Table**

**Table of Habitat-suitability rank and InVEST-sensitivity rank**. Rankings of National Agricultural Statistics Service (NASS) land-cover habitat categories by suitability as breeding habitat for grassland-bird species (Habitat column) in the Prairie Pothole Region of the United States, whereby habitat rankings are scored from 0 (unsuitable habitat) to 1 (most-suitable habitat). Rankings of NASS land-cover habitat categories by relative impact, or sensitivity, of those five land uses that are deemed threats to grassland-bird habitat integrity (5 Sensitivity columns) to the integrity of each individual land cover when they are adjacent to one another on the landscape. Sensitivity rankings are scored from 0 (low sensitivity) to 1 (highest sensitivity). Sensitivity rankings are input into Integrated Valuation of Ecosystem Services and Tradeoffs (InVEST) models (modeling suite version 3.2.0, Natural Capital Project 2015).

| **NASS Land-cover Category** | **Habitat** | **Sensitivity to Crop** | **Sensitivity to Energy** | **Sensitivity to Roads** | **Sensitivity to Urban** | **Sensitivity to Woodland** |
| --- | --- | --- | --- | --- | --- | --- |
| Grassland, Herbaceous | 1 | 0.75 | 0.6 | 0.6 | 0.9 | 0.9 |
| Conservation Reserve Program | 1 | 0.75 | 0.6 | 0.6 | 0.9 | 0.9 |
| Hayland | 0.9 | 0.75 | 0.6 | 0.6 | 0.9 | 0.9 |
| Alfalfa | 0.85 | 0.75 | 0.6 | 0.6 | 0.9 | 0.9 |
| Clover, Wildflowers | 0.85 | 0.75 | 0.6 | 0.6 | 0.9 | 0.9 |
| Switchgrass | 0.5 | 0.75 | 0.6 | 0.6 | 0.9 | 0.9 |
| Vetch | 0.5 | 0.75 | 0.6 | 0.6 | 0.9 | 0.9 |
| Barley | 0.5 | 0.75 | 0.6 | 0.6 | 0.9 | 0.9 |
| Dual Crop, Barley/Corn | 0.5 | 0.75 | 0.6 | 0.6 | 0.9 | 0.9 |
| Dual Crop, Barley/Sorghum | 0.5 | 0.75 | 0.6 | 0.6 | 0.9 | 0.9 |
| Dual Crop, Barley/Soybeans | 0.5 | 0.75 | 0.6 | 0.6 | 0.9 | 0.9 |
| Durum Wheat | 0.5 | 0.75 | 0.6 | 0.6 | 0.9 | 0.9 |
| Spring Wheat | 0.5 | 0.75 | 0.6 | 0.6 | 0.9 | 0.9 |
| Winter Wheat | 0.5 | 0.75 | 0.6 | 0.6 | 0.9 | 0.9 |
| Dual Crop, WtrWheat/Soy | 0.5 | 0.75 | 0.6 | 0.6 | 0.9 | 0.9 |
| Dual Crop, WtrWheat/Corn | 0.5 | 0.75 | 0.6 | 0.6 | 0.9 | 0.9 |
| Rye | 0.5 | 0.75 | 0.6 | 0.6 | 0.9 | 0.9 |
| Oats | 0.5 | 0.75 | 0.6 | 0.6 | 0.9 | 0.9 |
| Dual Crop, Oats/Corn | 0.5 | 0.75 | 0.6 | 0.6 | 0.9 | 0.9 |
| Millet | 0.5 | 0.75 | 0.6 | 0.6 | 0.9 | 0.9 |
| Other Small Grains | 0.5 | 0.75 | 0.6 | 0.6 | 0.9 | 0.9 |
| Fallow, Idle | 0.3 | 0.75 | 0.6 | 0.6 | 0.9 | 0.9 |
| Sorghum | 0 | 0 | 0 | 0 | 0 | 0 |
| Soybeans | 0 | 0 | 0 | 0 | 0 | 0 |
| Dual Crop, Soy/Soy | 0 | 0 | 0 | 0 | 0 | 0 |
| Dual Crop, Corn/Soy | 0 | 0 | 0 | 0 | 0 | 0 |
| Corn | 0 | 0 | 0 | 0 | 0 | 0 |
| Sweet Corn | 0 | 0 | 0 | 0 | 0 | 0 |
| Popcorn | 0 | 0 | 0 | 0 | 0 | 0 |
| Sunflower | 0 | 0 | 0 | 0 | 0 | 0 |
| Canola | 0 | 0 | 0 | 0 | 0 | 0 |
| Flaxseed | 0 | 0 | 0 | 0 | 0 | 0 |
| Safflower | 0 | 0 | 0 | 0 | 0 | 0 |
| Rape Seed | 0 | 0 | 0 | 0 | 0 | 0 |
| Mustard | 0 | 0 | 0 | 0 | 0 | 0 |
| Camelina | 0 | 0 | 0 | 0 | 0 | 0 |
| Buckwheat | 0 | 0 | 0 | 0 | 0 | 0 |
| Sugarbeets | 0 | 0 | 0 | 0 | 0 | 0 |
| Dry Beans | 0 | 0 | 0 | 0 | 0 | 0 |
| Potatoes | 0 | 0 | 0 | 0 | 0 | 0 |
| Onions | 0 | 0 | 0 | 0 | 0 | 0 |
| Cucumbers | 0 | 0 | 0 | 0 | 0 | 0 |
| Lentils | 0 | 0 | 0 | 0 | 0 | 0 |
| Peas | 0 | 0 | 0 | 0 | 0 | 0 |
| Caneberries | 0 | 0 | 0 | 0 | 0 | 0 |
| Herbs | 0 | 0 | 0 | 0 | 0 | 0 |
| Sod | 0 | 0 | 0 | 0 | 0 | 0 |
| Triticale | 0 | 0 | 0 | 0 | 0 | 0 |
| Carrots | 0 | 0 | 0 | 0 | 0 | 0 |
| Asparagus | 0 | 0 | 0 | 0 | 0 | 0 |
| Pumpkins | 0 | 0 | 0 | 0 | 0 | 0 |
| Peppers | 0 | 0 | 0 | 0 | 0 | 0 |
| Strawberries | 0 | 0 | 0 | 0 | 0 | 0 |
| Squash | 0 | 0 | 0 | 0 | 0 | 0 |
| Cabbage | 0 | 0 | 0 | 0 | 0 | 0 |
| Radishes | 0 | 0 | 0 | 0 | 0 | 0 |
| Turnips | 0 | 0 | 0 | 0 | 0 | 0 |
| Cranberries | 0 | 0 | 0 | 0 | 0 | 0 |
| Other Crops | 0 | 0 | 0 | 0 | 0 | 0 |
| Grapes | 0 | 0 | 0 | 0 | 0 | 0 |
| Apples | 0 | 0 | 0 | 0 | 0 | 0 |
| Miscellaneous Fruits, Vegetables | 0 | 0 | 0 | 0 | 0 | 0 |
| Almonds | 0 | 0 | 0 | 0 | 0 | 0 |
| Deciduous Forest | 0 | 0 | 0 | 0 | 0 | 0 |
| Evergreen Forest | 0 | 0 | 0 | 0 | 0 | 0 |
| Mixed Forest | 0 | 0 | 0 | 0 | 0 | 0 |
| Shrubland | 0 | 0 | 0 | 0 | 0 | 0 |
| Christmas tree | 0 | 0 | 0 | 0 | 0 | 0 |
| Temporary Wetland | 0 | 0 | 0 | 0 | 0 | 0 |
| Seasonal Wetland | 0 | 0 | 0 | 0 | 0 | 0 |
| Semi-Perm Wetland | 0 | 0 | 0 | 0 | 0 | 0 |
| Permanent Wetlands | 0 | 0 | 0 | 0 | 0 | 0 |
| Lacustrine Wetlands | 0 | 0 | 0 | 0 | 0 | 0 |
| Riverine Wetlands | 0 | 0 | 0 | 0 | 0 | 0 |
| Artificial/Other Wetlands | 0 | 0 | 0 | 0 | 0 | 0 |
| Woody Wetlands | 0 | 0 | 0 | 0 | 0 | 0 |
| Herbaceous Wetlands | 0 | 0 | 0 | 0 | 0 | 0 |
| Open Water | 0 | 0 | 0 | 0 | 0 | 0 |
| Developed Open | 0 | 0 | 0 | 0 | 0 | 0 |
| Developed Low | 0 | 0 | 0 | 0 | 0 | 0 |
| Developed Med | 0 | 0 | 0 | 0 | 0 | 0 |
| Developed High | 0 | 0 | 0 | 0 | 0 | 0 |
| Barren | 0 | 0 | 0 | 0 | 0 | 0 |
