# Supplementary material for: Modeling effects of crop production, energy development and conservation-grassland loss on avian habitat
Source: PLoS One. 2019 Jan 9;14(1):e0198382. doi: 10.1371/journal.pone.0198382 (PMC6326430; doi:10.1371/journal.pone.0198382)
Supplement: S4 Table — Land-use categories treated as threats to the integrity of grassland-bird habitat in the Prairie Pothole Region of the United States are organized by their relative threat value, or weight. Distance reflects how far an influence a pixel of a threat exerts on surrounding pixels. (DOCX) [file pone.0198382.s004.docx]

**Supporting Information**

**S4 Table**

**Table of InVEST threat rank and distance impact.** Land-use categories treated as threats to the integrity of grassland-bird habitat in the Prairie Pothole Region of the United States are organized by their relative threat value, or weight, whereby higher values reflect a higher threat. Distance reflects how far an influence a pixel of a threat exerts on surrounding pixels. Threat rank and distance impact are input into Integrated Valuation of Ecosystem Services and Tradeoffs (InVEST) models (modeling suite version 3.2.0, Natural Capital Project 2015).

| **Threat** | **Weight** | **Distance (km)** |
| --- | --- | --- |
| Woodland | 1 | 1.6 |
| Urban | 1 | 0.5 |
| Crop | 0.75 | 0.1 |
| Road | 0.5 | 0.5 |
| Energy | 0.5 | 0.1 |

**References**

Natural Capital Project, 2015. InVEST Version 3.2.0. (<http://www.naturalcapitalproject.org/invest/>. Accessed 4 December 2017).

United States Department of Agriculture (USDA), 2016. CRP Contract Summary and Statistics: Annual Summary. (<http://www.fsa.usda.gov/FSA/webapp?area=home&subject=copr&topic=crp-st/>. Accessed 4 December 2017).
