# Supplementary material for: Modeling effects of crop production, energy development and conservation-grassland loss on avian habitat
Source: PLoS One. 2019 Jan 9;14(1):e0198382. doi: 10.1371/journal.pone.0198382 (PMC6326430; doi:10.1371/journal.pone.0198382)
Supplement: S1 Fig — (DOCX) [file pone.0198382.s005.docx]

**Supporting Information**

**S1 Fig.**

Distribution of unsuitable habitat due to the impact of oil development in the Bakken Region of northwestern North Dakota, United States of America, showing the negative impact on habitat suitability of oil wells, the black squares.


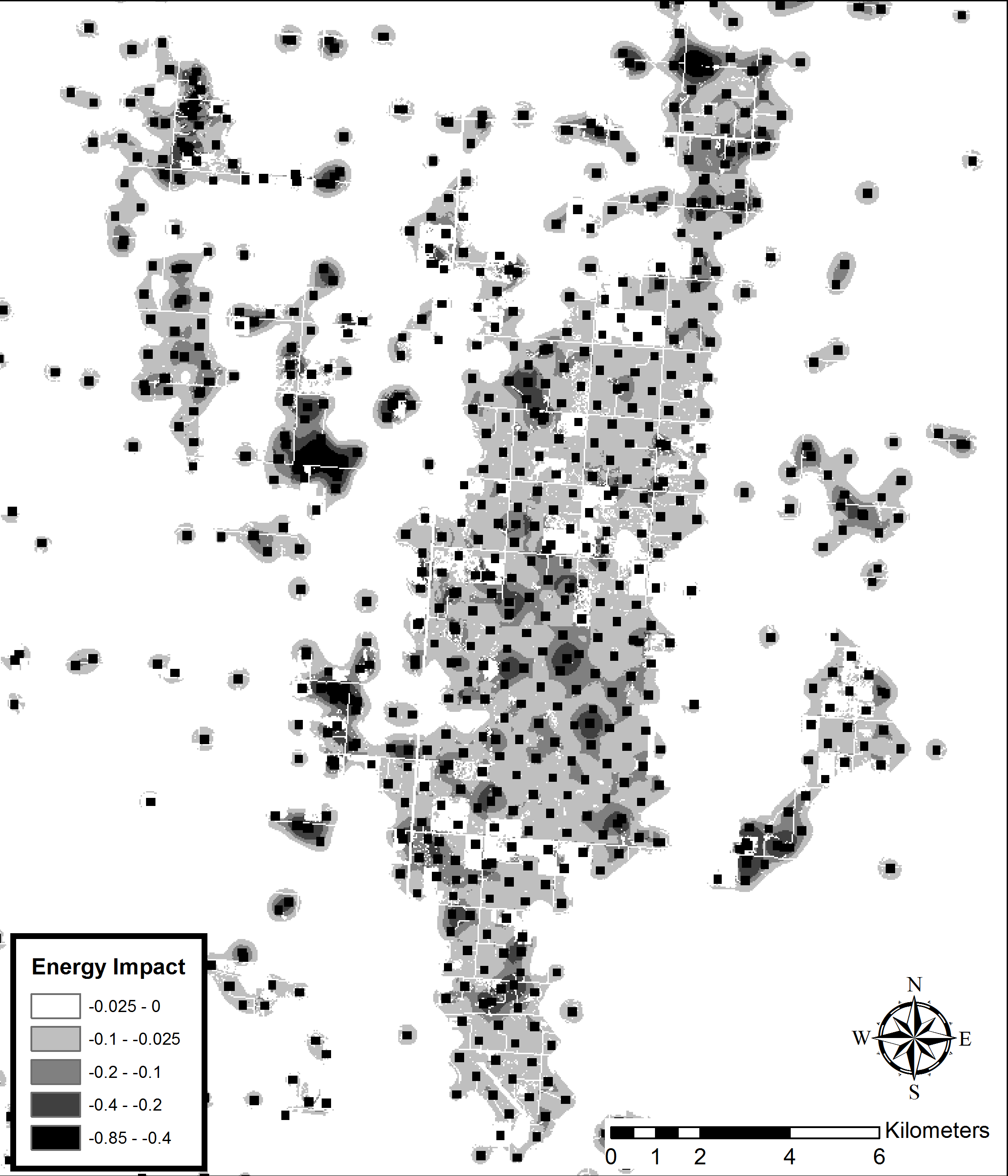


36 mi^2^

(93.2 km^2^)

Township
